# Factors Related to *Bacillus thuringiensis* and Gut Physiology. Comment on Rajan, V. An Alkaline Foregut Protects Herbivores from Latex in Forage, but Increases Their Susceptibility to Bt Endotoxin. *Life* 2023, *13*, 2195

**DOI:** 10.3390/life14020205

**Published:** 2024-01-31

**Authors:** Colin Berry

**Affiliations:** Cardiff School of Biosciences, Cardiff University, Cardiff CF10 3AX, UK; berry@cardiff.ac.uk

**Keywords:** *Bacillus thuringiensis*, pesticidal proteins, insecticidal toxins

## Abstract

A recent article has proposed that alkaline guts may lead to a general susceptibility to the biological control agent *Bacillus thuringiensis* and the pesticidal proteins derived from it. An analysis of the literature presented here clarifies our knowledge on the activity and safety of these agents, indicating that alkaline guts are not determinant of sensitivity and that the generalized conclusions proposed in the previous article cannot be substantiated.

## 1. Bt Insecticidal Proteins Are Not Detrimental to Most Invertebrates and Vertebrates

*Bacillus thuringiensis* (Bt) has been widely used in the field for more than 60 years and has a long history of safe usage. Since the mid-1990s, transgenic crops expressing Bt toxins for pest resistance have also been increasingly deployed—with the additional benefit of reduced usage of conventional broad-spectrum pesticides that have potential deleterious effects [1,2]. Extensive safety tests have been carried out in order to licence products for use and, in addition, a wide range of laboratory and field experiments have been performed to analyse factors such as host range, mechanisms of action, processing and activation, target specificity, receptor binding, off-target effects, insect resistance and safety. The overall message from this extensive literature is that Bt and products derived from it are safe, showing selectivity for a very limited number of targets [3,4]. Even within narrow taxonomic divisions, toxins are able to discriminate between individual species that they are able to affect and those that are refractory (see below). 

Against this background, the recent paper published in this journal by Rajan [5] proposes that alkaline foreguts in a range of herbivores may have evolved to deal with dietary latex and, at the same time, that this increases their susceptibility to endotoxins from *Bacillus thuringiensis* (Bt). Whether or not latex is likely to be a driver of gut adaptations will not be addressed here, except to note that a range of other factors may also underpin the evolution of physiological conditions in the gut. For example, adaptation of gut pH in a range of insects in the order Lepidoptera has been proposed as a response to the relative intake of dietary tannins [6]. Beyond proposals linked to latex in diets, the Rajan paper also makes a number of general statements about the susceptibility of alkaline midguts to delta-endotoxins derived from Bt and other bacteria. Statements generalising about Bt toxins are hard to justify and should be avoided. Bt toxins belong to at least 10 distinct structural classes (plus proteins currently classed under the holding name Xpp since structural data are not available at present [7]). Even if the consideration is limited to the proteins produced as natural crystals, a wide variety of proteins are also encompassed (App, Cyt, Cry, Gpp, Mpp, Tpp structural classes and Xpp proteins). This means that general statements cannot be substantiated across this range of proteins and their different mechanisms of action. Thus, Rajan’s hypotheses lack details and are overly simplistic.

While there are some minor errors in the description of toxins (the Mtx toxins of *Lysinibacillus sphaericus*, now classified as either Mtx or Mpp proteins [7], are mischaracterised as sporulation, rather than vegetatively produced proteins), the major concern with the paper is the number of statements and generalisations that imply a general susceptibility of animals with alkaline foreguts to Bt and its toxins and implications for safety. A review of the literature shows that these proposals are not correct.

## 2. Bt Susceptibility Does Not Tell Us about Gut pH

The reader is told that “*While direct measurement may be difficult in very small animals, there is an indirect way to test the pH of the first chamber of the gut by measuring the organism’s susceptibility to Bacillus thuringiensis crystal δ-endotoxin (Bt)*”. Again, this is clearly incorrect. Larvae of most Lepidoptera and many Diptera have alkaline guts, yet a given Bt toxin will kill only a narrow subset of these insects. There are many instances where Bt proteins are active in some alkaline insect guts but not in others, e.g., Cry9Bb1 kills *Manduca sexta* but not *Mamestra brassicae* [8] and the gut pH in both of these Lepidoptera, belonging to the same taxonomic clade, is pH 9.6 [6]. Thus, sensitivity to Bt Cry proteins cannot be used as an indicator of gut pH.

## 3. Gut pH Cannot Predict Sensitivity to Bt Proteins or Susceptibility to Bt Colonization

Rajan suggests that an alkaline gut renders animals susceptible to toxins produced by Bt. While many Bt targets do have alkaline guts [those in the Diptera suborder Nematocera (mosquitoes and midges) and Lepidoptera (butterflies and moths)], many targets also have acidic guts or near-neutral guts. For example, dipterans of the suborders Brachycera (horse flies and hover flies) and Cyclorrhapha (house flies [9], blowflies, fruit flies [10]) have guts that are neutral-to-slightly acidic, particularly in the foregut regions. Diptera with acidic foreguts such as *Musca domestica* have a documented susceptibility to Cry1Ba1 [11] and have a gut pH that ranges from 6.1 (anterior) through 3.1 (middle) to 6.8 (posterior) [9]. Other examples include Coleoptera from Tenebrionidae, such as *Tribolium castaneum,* which is susceptible to the Bt proteins Mpp23/Xpp37 [12] and has a foregut–midgut–hindgut pH profile of 5.2, 7.2–7.6 and 3.6–4.6 [13]; *Tenebrio molitor*, which is susceptible to the Bt protein Cry3Aa [14] and has a gut pH ranging from 5.2 to 8.2 anterior to posterior [15]; the crysomelid, *Diabrotica vergifera vergifera*, which is susceptible to Cry1Bh1 [16], Cry3b [17] App6Aa1 and Gpp34/Tpp35 [18] and has a gut pH of 5.75 [19]; *Leptinotarsa decemlineata*, which is susceptible to Cry3Aa and Cry3Ab [20], Cry7Aa2 [21], Mpp51Aa1 [22] and Mpp51Aa2 [23] and has a foregut–midgut–hindgut pH profile of 5.9, 5.9–6.6 and 6.5 [13]. Beyond insects, the nematode *Caenorhabditis elegans* is susceptible to App6Aa1, Cry5Ba1, Cry21Aa1 [24], Cry21Fa1, Cry21Ha1 [25], Cry5Ca1 and Cry5Da1 [26] and this nematode has a gut pH ranging from 5.96 +/− 0.31 in the anterior pharynx to 3.59 +/− 0.09 in the posterior intestine [27]. We must conclude that gut pH is not a predictor of pesticidal protein sensitivity.

## 4. Alkaline Solubilisation of Bt Toxins Is Only Part of the Story

The Rajan paper also seeks to draw a link between a necessity for alkaline solubilisation of crystal toxins and their subsequent toxicity. This, too, gives an incomplete picture of the situation. Where Rajan refers to the use of Bt pesticidal proteins in transgenic plants, the alkaline environment of the gut has no relevance for toxin solubilisation as the proteins are expressed in mature, soluble forms and not as crystals (if the role of alkalinity was simply for solubilisation, there would be potential for the expression in soluble form to make many non-targets more sensitive, but there is no evidence that any increased sensitivity is induced). In addition, for toxins produced as crystals and applied with the spores of their host Bt strains, alkaline conditions may not be the only route to solubilisation. The nematocidal toxin App6Aa2 is soluble in both alkaline and acid conditions [28], consistent with its activity in the acidic guts of *D. vergifera vergifera* and *C. elegans*. Similarly, the Cry3A protein, which is active against *L. decemlineata,* is soluble at either acid or alkaline pH, with proteolysis assisting in acid solubility [29]. Therefore, from the above, it can be seen that an acidic foregut does not, of itself, preclude either crystal protein solubilisation or invertebrate toxicity.

## 5. Alkaline Foreguts Do Not per se Render Animals Vulnerable to Bt Toxins

Most concerning are the assertions in Rajan’s paper that “*the presence of an alkaline gut pH makes foregut-fermenting mammals, metamorphosing tadpoles, and certain orders of insects susceptible to gut damage by Bt and related insecticidal toxins*” and “*an alkaline gut, however, renders animals susceptible to the action of toxins produced by Bacillus thuringiensis and Lysinibacillus sphaericus*”. Alkaline guts alone are, clearly, not sufficient to induce host sensitivity, as evidenced above. A critically important oversight of Rajan’s assertions are that he ignores the vast literature that shows receptor dependence for pesticidal proteins [30] and that mutation of receptors can render normally susceptible species resistant (e.g., [31,32]). This renders animals lacking receptors, like most invertebrates and vertebrates, insensitive to the toxins. In reality, Bt pesticidal proteins kill only a narrow range of targets. Moreover, this level of selectivity can mean that even insects within a small taxonomic subgroup can show variable susceptibility. For example, Cry4Ba is toxic against the mosquitoes *Aedes aegypti* and *Anopheles stephensi* but not *Culex pipiens* [33]; Cry48Aa1/Tpp49Aa1 are toxic to *Culex quinquefasciatus, An. stephensi* and *Aedes albopictus* but not against *Anopheles gambiae* or *Ae. aegypti* [34,35]; Tpp1/Tpp2 kill *Aedes atropalpus* but have very limited to no effect against *Ae. aegypti* [36]. Beyond receptors, the proteinase susceptibility of individual toxins, along with the proteinase arsenal of the gut, are also important factors, independent of whether the gut is acidic or alkaline. Thus, Cry3 proteins degrade rapidly in simulated gastric juice [37] but are still able to exert toxicity in the acidic guts of target beetles, and changes in proteinase activity can lead to reduced Cry3Aa susceptibility [38]. Some individual toxins may also be proteolytically activated in different ways with consequent effects on target range [39].

The extent of safety tests is also brought into question, stating that tests were carried out on animals such as rats and mice, but ignoring the copious other tests and exposures that have been assessed. Mammalian safety tests on Bt products have a long history [40] and, contrary to what is stated in the publication, their effects on ruminants have been analysed. For example, with respect to ungulate mammals with alkaline foreguts, testing of Bt strains in direct feeding to sheep found no adverse consequences [41,42].

As a result, the safety risks of Bt and its toxins implied by Rajan are simply not upheld given the evidence available. It is important to note the use of genetically modified crops expressing Bt proteins (particularly maize and soybeans) has increased since their first approval in 1995 [43] to the point that approximately 80% of the areas cultivated for both corn and cotton in the USA are sown with Bt products [44]. These crops have regularly been used as primary feeds for poultry, pigs, dairy and beef cattle in North America and Brazil for over 20 years, yet no adverse effects have been reported [45,46]. Use as feeds on such a scale in these countries would certainly have been expected to show significant effects if such effects existed in these major markets. The papers cited by Rajan to support the suggestion that Bt may have effects on cattle include an opinion-based questionnaire survey that has no direct and rigorous comparison of Bt-GM-fed animals with controls fed on non-GM material [47], a study that specifically and explicitly states in both the abstract and the first line of the introduction that “*This study was not designed as a scientific experiment*” [48], and what appears to be a non-peer reviewed conference report of circumstantial associations of effects on livestock with no strict controls and, in many of the instances listed in the report, the author is clear that it was impossible under the survey conditions to tell whether other conditions, including blue tongue and Peste du petits ruminants, were responsible for animal health issues [49]. Given the paucity of evidence for adverse effects, the questioning of Bt safety “*making Bt off-target impact and consequential ecological safety questionable*” is most certainly unfounded and unhelpful as we try to maintain and increase crop yields while moving away from broad-host-range chemical pesticides.
